# The Combined Antibacterial Mechanism of Ferulic Acid and ε-Polylysine Hydrochloride in *Shewanella putrefaciens* and the Effect of Their Application on the Storage Quality of Refrigerated Crayfish (*Procambarus clarkii*) with Plasma-Activated Water

**DOI:** 10.3390/foods14111942

**Published:** 2025-05-29

**Authors:** Yue Cui, Tengteng Zhang, Dandan Zhao, Sai Gao, Yinchu Liu, Xinyu Yang, Han Lu, Xiaoguang Gao

**Affiliations:** College of Food Science and Biology, Hebei University of Science and Technology, No. 26 Yuxiang Street, Yuhua District, Shijiazhuang 050000, China; cuiy1223@hotmail.com (Y.C.); ztteng8364@163.com (T.Z.); zdd6364@126.com (D.Z.); gaosai080911@163.com (S.G.); liuyinchu88@163.com (Y.L.); xinyuyang1127@126.com (X.Y.); jingmaoluhan@126.com (H.L.)

**Keywords:** crayfish, *Shewanella putrefaciens*, ferulic acid, ε-polylysine hydrochloride, plasma-activated water

## Abstract

This study aimed to investigate the mechanism underlying the synergistic antimicrobial effect of ferulic acid (FA) and ε-polylysine hydrochloride (PL) on Shewanella putrefaciens (*S. putrefaciens*) and their application on crayfish (*Procambarus clarkii*). The treatment with FA and PL exhibited a strong synergistic inhibitory effect against S. putrefaciens. The combination of 1/4 Minimum Inhibitory Concentration (MIC) FA and 1/4 MIC PL was the most effective, damaging the cell structure and inhibiting the growth of *S. putrefaciens*. Plasma-activated water (PAW) can induce microbial inactivation through physical action. In addition, treatments with FA, PL, PAW, PAW-FA, PAW-PL, and PAW + PL-FA substantially decreased total viable counts (TVCs), total volatile base nitrogen (TVB-N), the thiobarbituric acid value (TBA), and the juice loss rate of crayfish, with FA-PL showing the best effect. This study confirmed the antimicrobial efficacy of PL, FA, and PAW, indicating their potential as effective preservatives for controlling spoilage in freshwater crustaceans.

## 1. Introduction

Aquatic products are rich in nutrients and have a high moisture content, making them susceptible to microbial growth during storage and transport, which can lead to spoilage [1,2,3]. Crayfish (*Procambarus clarkii*) are freshwater crustaceans that belong to the Astacoidea and Parastacoidea superfamilies [4]. Crayfish is a significant economic freshwater product in China, cherished by consumers for its delectable flavor and convenience [5]. Crayfish are abundant in nutrients such as protein and lipids [6,7]. *Shewanella putrefaciens* (*S. putrefaciens*) is among the Gram-negative specific spoilage organisms (SSOs) of aquatic products [3,8]. It enzymatically breaks down proteins and fats, converting trimethylamine oxide into foul-smelling compounds [9,10]. Therefore, inhibiting *S. putrefaciens* would be advantageous in improving the quality of aquatic products.

ε-Polylysine hydrochloride (PL) is derived from the fermentation metabolites of *Streptomyces diastatochromogenes*. It is a homopolymer of the natural polypeptide ε-polylysine, comprising 25 to 35 L-lysine residues [11,12]. PL exhibits a wide range of bacteriostatic properties but has a limited impact on certain types of spoilage bacteria and food sensory attributes [13]. PL has been recognized as a food additive in China since 2014 and is used commercially in various countries [14,15]. However, limited information is available regarding the potential application and effects of PL on the growth and thermal inactivation of *S. putrefaciens*.

Combining synergistic natural antimicrobial substances has shown greater efficacy than using them individually, improving both antibacterial effectiveness and sensory palatability [11,16]. Ferulic acid (4-hydroxy-3-methoxycinnamic acid, FA) has gained attention due to its antioxidant properties, and it occurs naturally in various plants, such as vegetables, coffee, nuts, and cereals [17,18]. However, the synergistic antibacterial activity of phenolic acids and PL has not been assessed and requires confirmation through antibacterial testing.

The application of plasma-activated water (PAW) as an aqueous disinfectant in the food industry has garnered increasing attention [19,20]. During PAW preparation, active nitrogen, active oxygen, and other active ingredients are possibly formed in the liquid, contributing to the bactericidal activity in aquatic products. A study targeting white shrimp showed that, compared with tap water ice, PAW ice had a significant advantage in inhibiting microbial growth, which can delay the deterioration of color and hardness in crayfish [21].

**Figure 1:**
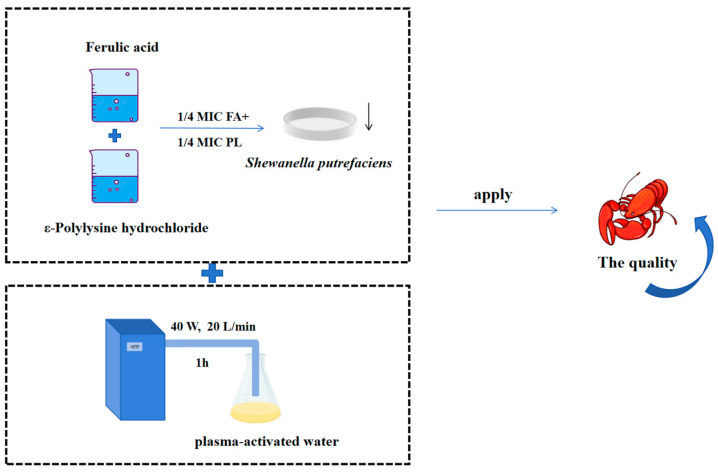
The schematic graphic of the experiment. The upper section depicts the screening of FA and PL, while the lower section illustrates the preparation of plasma-activated water. The objective is to observe its effect on the quality of crayfish.

The study used a twofold dilution method to determine the MIC and the fractional inhibitory concentration index (FICI) of FA and PL against the dominant spoilage bacterium in aquatic products. The combined inhibitory mechanisms of FA and PL against *S. putrefaciens* were preliminarily investigated with respect to cell wall integrity, cell membrane permeability, bacterial micromorphology, and nucleic acid synthesis. Subsequently, since crayfish contained a variety of spoilage bacteria, not limited to *S. putrefaciens*. FA, PL, and PAW were used to investigate their impact on the quality changes in crayfish during refrigeration, thereby assessing the overall effectiveness of the treatment (Figure 1).

## 2. Materials and Methods

### 2.1. Chemicals and Bacterial Strains

FA and PL were purchased from Aladdin Biochemical Technology Co., Ltd. (Beijing, China). N-Phenyl-1-naphthylamine (NPN) and propidium iodide (PI) were purchased from Yuanye Bioengineering Institute Co., Ltd. (Beijing, China). Marker, DNA Ladder, and phosphate-buffered saline (PBS) were purchased from Solarbio Co., Ltd. (Beijing, China). Sodium chloride and agar powder were purchased from Yongda Chemical Reagent Co., Ltd. (Beijing, China). The *S. putrefaciens* (CICC 22940) was procured from the China Center for Industrial Culture Collection (CICC) and stored at −80 °C. All other reagents were commercial analytical grade.

### 2.2. Antibacterial Test of PL and FA

#### 2.2.1. Determination of MIC Values

The broth micro-dilution method was employed to determine the MIC of PL and FA [22]. The bacterial suspension of *S. putrefaciens* was diluted to 10^7^ CFU/mL after inoculation in Tryptic Soy Broth (TSB) and incubation at 30 °C, reaching the logarithmic phase. In a 96-well polystyrene microtiter plate, the PL solution was diluted to obtain 9 different concentrations (10 mg/mL, 5 mg/mL, 2.5 mg/mL, 1.25 mg/mL, 0.625 mg/mL, 0.313 mg/mL, 0.157 mg/mL, 0.0781 mg/mL, 0.0391 mg/mL) by TSB broth medium. The FA solution underwent serial dilution in a 96-well plate using TSB broth medium to generate nine concentration gradients: 6 mg/mL, 3 mg/mL, 1.5 mg/mL, 0.75 mg/mL, 0.375 mg/mL, 0.188 mg/mL, 0.093 mg/mL, 0.047 mg/mL, and 0.023 mg/mL. Controls included TSB without bacteria and a bacterial suspension incubated without antimicrobials. The plates were incubated at 30 °C for 24 h. Subsequently, the absorbance at OD_600_ was measured using a microplate reader (SpectraMax I3x, Molecular Devices, Shanghai, China).

#### 2.2.2. Determination of the FICI Values

The checkerboard method devised by Cao et al. [23] was used to determine the FICI values for the combination of FA and PL. Serial 2-fold dilutions of FA and PL were made in a 96-well plate, ranging from 1/32 MIC to 4 MIC to obtain different concentration combinations. The *S. putrefaciens* suspension was adjusted to 10^7^ CFU/mL per well and incubated at 30 °C for 24 h. The FICI was calculated following Formula (1):

FICI = MIC_(A/B)_/MIC_A_ + MIC_(B/A)_/MIC_B_ (1)

MIC_A_ is the MIC of FA, and MIC_B_ is the MIC of PL. For the interpretation of the results, an FICI ≤ 0.5 indicates a synergistic effect of FA and PL combinations.

#### 2.2.3. Time-Killed Curve Plotting

An inoculum of *S. putrefaciens* suspension, cultured to the logarithmic phase, was introduced into 100 mL of TSB suspension with varying concentrations of preservatives (0, MIC FA, MIC PL, FA, 1/4 MIC PL, 1/4 MIC FA + 1/4 MIC PL) to achieve a concentration of 10^7^ CFU/mL. The plates were then incubated at 30 °C in a constant-temperature incubator. At predetermined intervals, a specific volume of bacterial suspension was sampled to assess the viable bacterial count.

#### 2.2.4. Determination of Alkaline Phosphatase (AKP) Activity

The *S. putrefaciens* suspension in the logarithmic phase (10^7^ CFU/mL) was centrifuged, and the sedimented bacteria were washed twice with saline. Different concentrations of preservatives (MIC FA, MIC PL, 1/4 MIC FA, 1/4 MIC PL, 1/4 MIC FA + 1/4 MIC PL) were added. After 6 h of incubation in a thermostatic water bath at 30 °C, the samples were centrifuged to collect bacterial cells. The bacterial suspensions were then subjected to ultrasonic crushing in a 400 W ice water bath for 5 min until the suspension was clarified. After centrifugation, the supernatant was collected for the determination of intracellular alkaline phosphatase activity using an Alkaline Phosphatase Assay Kit (Jiancheng Bioengineering Co., Ltd., Jiangsu, China).

#### 2.2.5. Determination of Extracellular Membrane Permeability

An N-phenyl-1-naphthylamine (NPN) fluorescent probe uptake assay was used to investigate the permeability of the outer membrane of *S. putrefaciens* pombe. NPN was obtained using the previous process with modifications [24]. After preservative treatment for 6 h, the samples were centrifuged (6000 rpm/min, 4 °C, 10 min) and resuspended in 1 mL of physiological saline. Then, 20 μL of NPN (0.5 mmol/L) was added, and the mixture was incubated in the dark at 25 °C for 10 min. Thereafter, fluorescence was quantified at an excitation wavelength of λex = 350 nm and an emission wavelength of λem = 429 nm.

#### 2.2.6. Propidium Iodide (PI) Uptake

PI was used to evaluate the cell membrane permeability of *S. putrefaciens* [25]. The samples were treated with the preservative for 6 h. The cells were suspended in 1 mL of physiological saline, and 100 μL of PI (100 μg/mL) staining solution was added. The mixture was then incubated for 15 min at 37 °C in the dark. Fluorescence was quantified at an excitation wavelength of 482 nm and an emission wavelength of 635 nm. The fluorescence intensity was measured and observed using a fluorescent inverted microscope, with images captured (Axio Imager.A2, Carl Zeiss AG, Shanghai, China).

#### 2.2.7. Leakage of Intracellular K^+^

Refer to Section 2.2.4 for the pre-treatment of the sample. After treating the samples with preservatives for 6 h, they were centrifuged at 6000 rpm for 10 min. The supernatant was collected, and the Potassium Assay Kit (Jiancheng Bioengineering Co., Ltd., Nanjing, China) was used to determine the potassium content of *S. putrefaciens* according to the provided instructions.

#### 2.2.8. Cell Morphology Observation

Scanning electron microscopy (SEM) analysis was performed following a previously reported method [26]. Refer to Section 2.2.4 for the pre-treatment of the sample. The cells were fixed with 2.5% glutaraldehyde solution for 12 h at 4 °C and rinsed with PBS three times. The cell pellets were dehydrated in ethanol solutions of increasing concentrations (30%, 50%, 70%, 85%, 90%, and 100%) for 15 min each and washed twice with isopentyl acetate for 20 min each time. Finally, the samples were collected by centrifugation, lyophilized, gold-sprayed, and observed using scanning electron microscopy (FEI, Hillsboro, OR, USA).

#### 2.2.9. DNA Extraction

The DNA extraction was conducted according to a previous report with slight modification [27]. *S. putrefaciens* was cultured to the log phase, and its genomic DNA was extracted using a Bacterial Genomic DNA Extraction Kit (Solarbio, Co., Ltd., Beijing, China). The mixture was subjected to electrophoresis on agarose gel (1%, 100 V, 45 min) and visualized using a gel imaging system (Gel Doc XR+, Bio-rad, Hercules, CA, USA).

### 2.3. Application of PL and FA in Crayfish

#### 2.3.1. Preparation and Treatment of Crayfish

Crayfish were prepared as described by Tang et al. [5]. Live crayfish (20 g ± 2.5 g body weight, approximately 240 individuals) were purchased from the Shiping Breeding Base (Baoding, China). After being immersed in crushed ice and suffocated to death, the heads were removed, the shells peeled, the crayfish cleaned, and the water drained. The tail meat of the crayfish was collected. These prepared crayfish served as the experimental samples.

All the crayfish were randomly divided into eight groups (approximately 30 in each group): (1) CK: control check; (2) FA: MIC FA treatment; (3) PL: MIC PL treatment; (4) PL-FA: 1/4 MIC FA combined with 1/4 MIC PL treatment; (5) PAW: PAW treatment; (6) PAW-FA: PAW combined with 1/4 MIC FA treatment; (7) PAW-PL: PAW combined with 1/4 MIC PL treatment; (8) PAW + PL-FA: PAW combined with 1/4 MIC FA and 1/4 MIC PL treatment. The crayfish were immersed in their respective solutions for 20 min, then stored in sterile sealed bags and refrigerated at 4 °C. The indicators for each group of crayfish were measured on days 1, 2, 3, 5, and 7 to assess freshness.

#### 2.3.2. Preparation of Low-Temperature Plasma-Activated Water (PAW)

A low-temperature plasma dry and wet preservation processor (SY-JXDW-01, Suzhou Fengyuanbao Agricultural Technology Co., Ltd., Suzhou, China) was used to prepare PAW. In this process, 100 mL of distilled water was placed in a beaker containing crushed ice. The distance between the preparation rod and the distilled water level was set to 1 cm, with a power setting of 40 W, a flow rate of 20 L/min, and a processing time of 1 h.

#### 2.3.3. Determination of Color Difference

The color parameters of crayfish were measured on the 2nd to 3rd abdominal segments of the tail using a portable colorimeter (YS2580, Sanen Times Technology Co., Ltd., Shenzhen, China). Prior to measurement, the instrument was calibrated using standard black and white reference plates. The recorded colorimetric values included lightness (L*), red–green chromaticity (a*), and yellow–blue chromaticity (b*).

#### 2.3.4. Total Viable Counts (TVCs)

Total viable counts were determined using the pour plate method on plate count agar (PCA), according to method of Wang et al. [28]. Ten g of crayfish flesh was homogenized in 18 mL of sterile water using a food homogenizer (FSH-2, Mengte Co., Ltd., Xianmen, China). The samples were incubated in a constant temperature incubator at 30 °C for 72 ± 3 h. The results were expressed as colony-forming units (CFU).

#### 2.3.5. Total Volatile Base Nitrogen (TVB-N) Value

The total volatile base nitrogen was measured according to the method of Chen et al. [29]. The 5 g sample was homogenized with 50 mL of distilled water using an electric mixer homogenizer. Then, the TVB-N was determined in the semi-automatic Kjeldahl Apparatus (Haineng Technology Instrument Co., Ltd., Jinan, China).

#### 2.3.6. Determination of pH

The treated sample (2 g) was mixed with 20 mL of distilled water, followed by homogenization and centrifugation (4500 rpm, 4 °C, 8 min). The supernatant was collected, and its pH was measured using a pH meter (ST2200ZH, Aohaosi Co., Ltd., Shanghai, China) equipped with a calibrated acidimeter. Each sample was repeated 3 times, and the results were averaged.

#### 2.3.7. Determination of Juice Loss of Meat

The previous method determined the juice loss of meat with minor modifications. The weight of crayfish was measured before refrigeration. Subsequently, the crayfish were removed at intervals according to the specified number of days. The surface moisture of the crayfish in each treatment group was absorbed with filter paper, and they were then placed on an electronic balance for weighing (ME 104/02, METTLER TOLEDO Co., Ltd., Shanghai, China). The juice loss of the meat was calculated following Formula (2):

Juice loss of Meat (%) = (m_1_ − m_2_)/m_1_ × 100 (2)

m_1_ is the quality of the crayfish before refrigeration, and m_2_ is the quality of the crayfish after refrigeration.

### 2.4. Statistical Analysis

All sampling was conducted a minimum of three times. The data are presented as mean values ± standard deviation (SD), and statistical analysis was performed using SPSS 26.0 (SPSS, IBM, Chicago, IL, USA). One-way ANOVA followed by Duncan’s multiple range test was used to determine significant differences (*p* < 0.05) between the means. All figures were created using Origin 2019.

## 3. Results and Discussion

### 3.1. Synergistic Antibacterial Effect of FA and PL

The MIC and FICI of FA and PL were used to determine the combined inhibitory effect of FA and PL on *S. putrefaciens*. The MIC FA and MIC PL were 3.0 mg/mL and 0.156 mg/mL, respectively. The FICI of the two in combination was 0.5 (1/4 + 1/4). Based on the experimental results, the combination of 1/4 MIC FA and 1/4 MIC PL was chosen for further experiments due to their synergistic effect.

**Figure 2:**
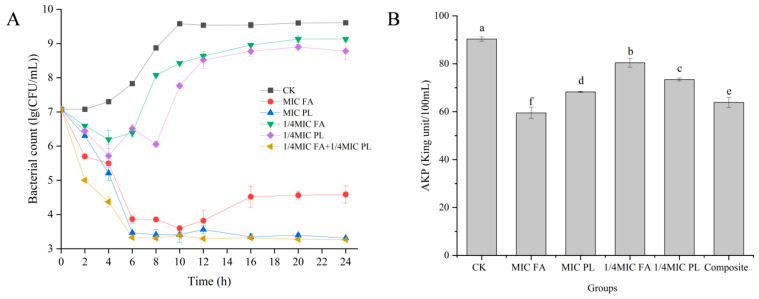
Time–kill curves of *S. putrefaciens* treated with FA and PL at 24 h (**A**). The effect of FA and PL on the activity of AKP in *S. putrefaciens* (**B**). Different lowercase letters (a, b, c, d, e, and f) indicate significant differences in AKP activity among the six different groups (*p* < 0.05). The data are expressed as mean values ± standard deviation (SD).

The inhibitory effects of FA, PL, and their combination on *S. putrefaciens* were further assessed by time–kill curves (Figure 2A). The inhibitory effect of 1/4 MIC FA and 1/4 MIC PL after 24 h of treatment was similar to that of the control group without the addition of preservative. In the 1/4 MIC FA + 1/4 MIC PL group, the number of colonies of *S. putrefaciens* decreased significantly before 6 h, and stabilized after 6 h. This effect was similar to the inhibitory effects of MIC FA (2.490 log_10_ CFU/mL) and MIC PL (3.766 log_10_ CFU/mL). The results showed that 1/4 MIC FA and 1/4 MIC PL alone could not inhibit the growth of *S. putrefaciens*. In comparison, the combined use of 1/4 MIC FA + 1/4 MIC PL achieved the bactericidal effect of a single preservative, MIC, and the number of colonies in the combined group was lower than that of the group treated with MIC FA and MIC PL. Li et al. [11] found that when hydrochloride compounds were tested for their synergistic antibacterial effects with gallic acid, they completely inhibited the growth of *S. putrefaciens* and caused cell death.

### 3.2. Effect of FA and PL on the S. putrefaciens Cell Wall

The cell wall is a critical structural component that protects the cell from foreign macromolecules and maintains its shape and mechanical strength [11]. Alkaline phosphatase (AKP) is mainly present between the cell wall and membrane. AKP leaks out from the wall membrane when the cell wall is damaged [30]. Therefore, changes in the AKP content of the organism can indicate cell wall disruption. The results shown in Figure 2B indicated that the control group had the highest AKP activity at 6 h. The AKP activity of *S. putrefaciens* treated with MIC FA and MIC PL was significantly reduced (*p* < 0.05), indicating that both FA and PL could disrupt the integrity of the cell wall of *S. putrefaciens*, leading to the leakage of bacterial AKP. These findings suggested that the combination of FA and PL significantly augmented the disruption of cell wall integrity in *S. putrefaciens*, indicating a synergistic effect between the two compounds.

### 3.3. Effect of FA and PL on the Permeability of Cell Membranes of S. putrefaciens

#### 3.3.1. Influence of Extracellular Membrane Permeability

**Figure 3:**
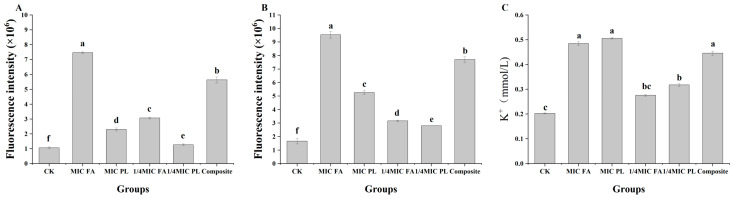
Effect of FA and PL on the permeability of the extracellular membrane (**A**). Effect of FA and PL on the fluorescence intensity of PI (**B**). Effect of FA and PL on the leakage of K^+^ (**C**). Different lowercase letters (a, b, c, d, e, and f) denote significant differences at *p* < 0.05 among the six groups of the same type. The data are expressed as mean values ± standard deviation (SD).

N-phenyl-1-naphthylamine (NPN) is a hydrophobic fluorescent probe that enhances its fluorescence intensity in non-polar or hydrophobic environments [26]. As shown in Figure 3A, each treatment resulted in increased NPN, suggesting that both FA and PL disrupted the extracellular membrane of *S. putrefaciens*. Furthermore, FA exhibited a more vital ability to disrupt the extracellular membrane than PL. In addition, the combination of 1/4 MIC FA and 1/4 MIC PL exhibited greater efficacy in disrupting the extracellular membrane of *S. putrefaciens* compared to MIC PL alone. This result suggested that the combination of 1/4 MIC FA and 1/4 MIC PL enhances the destructive effect of PL on the extracellular membrane of *S. putrefaciens*, resulting in a synergistic inhibitory outcome. The combined effect of PL and phenolic acids was significant compared to when each preservative was used alone, showing better efficacy than each on its own [11].

#### 3.3.2. Propidium Iodide (PI) Uptake

PI can be used to evaluate the permeability of the cytoplasmic membrane [31]. PI can enter cells with damaged cytoplasmic membranes, where it binds to the double-stranded structure of the DNA molecule, which then fluoresces red upon excitation. The PI uptake test of 1/4 MIC FA and 1/4 MIC PL, as well as their combination, demonstrated a significant increase in the absorption of the fluorescent nucleic acid stain after 6 h of exposure to antimicrobial agents compared to the control (Figure 3B). The fluorescence intensity after FA and PL treatment increased with the concentration of FA and PL. Individual MIC FA exhibited a lower rate of increase in fluorescence intensity compared to MIC PL. The fluorescence intensity observed in the combination groups of 1/4 MIC FA and 1/4 MIC PL was significantly greater than that in the individual treatment groups, indicating a synergistic effect.

**Figure 4:**
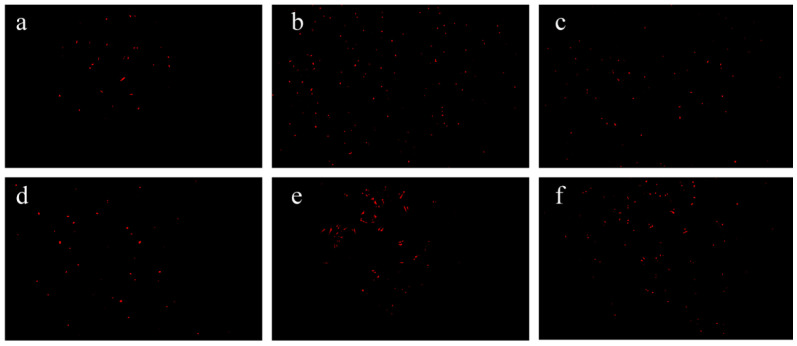
Effect of FA and PL on the permeability of cell membranes and apoptosis. (**a**): Control group; (**b**): MIC FA; (**c**): MIC PL; (**d**): 1/4 MIC FA; (**e**): 1/4 MIC PL; (**f**): 1/4MIC FA + 1/4 MIC PL.

Fluorescence inverted microscopy can be used to qualitatively assess the effects of FA and PL on the permeability of the cytoplasmic membrane of *S. putrefaciens* [24]. As shown in Figure 4a, less red fluorescence was observed, indicating that the cytoplasmic membrane remained intact. Treated with MIC FA, MIC PL, and the combination of 1/4 MIC FA and 1/4 MIC PL, *S. putrefaciens* exhibited extensive red fluorescence emission, as shown in Figure 4b–f, indicating significant disruption in the permeability of the cytoplasmic membrane. The synergistic effect was the most pronounced, and the combination of citral and carvacrol also demonstrated a strong bactericidal effect [23]. This finding aligns with the fluorescence intensity data presented earlier (Figure 2B), further supporting the synergistic action of the combination of 1/4 MIC FA and 1/4 MIC PL in disrupting the intracellular membranes of *S. putrefaciens*. These results indicate that the disruption of cytoplasmic membranes could account for the microbial inactivation induced by 1/4 MIC FA and 1/4 MIC PL [24].

#### 3.3.3. K^+^ Analysis

Figure 3C shows the effects of different preservatives on the leakage of potassium (K^+^) ions from the *S. putrefaciens* in 6 h. Leakage of K^+^ occurred to varying degrees using differently treated *S. putrefaciens*, with the K^+^ in the control supernatant being 0.203 mmol/L. The K^+^ leakage in the combination of 1/4 MIC FA and 1/4 MIC PL (0.446 mmol/L) was higher than with 1/4 MIC FA (0.276 mmol/L) and 1/4 MIC PL (0.318 mmol/L). The K^+^ leakage was similar to that of MIC FA, suggesting a synergistic effect of the combination on *S. putrefaciens* cells (*p* < 0.05).

### 3.4. Effect of FA and PL on Cell Morphology

**Figure 5:**
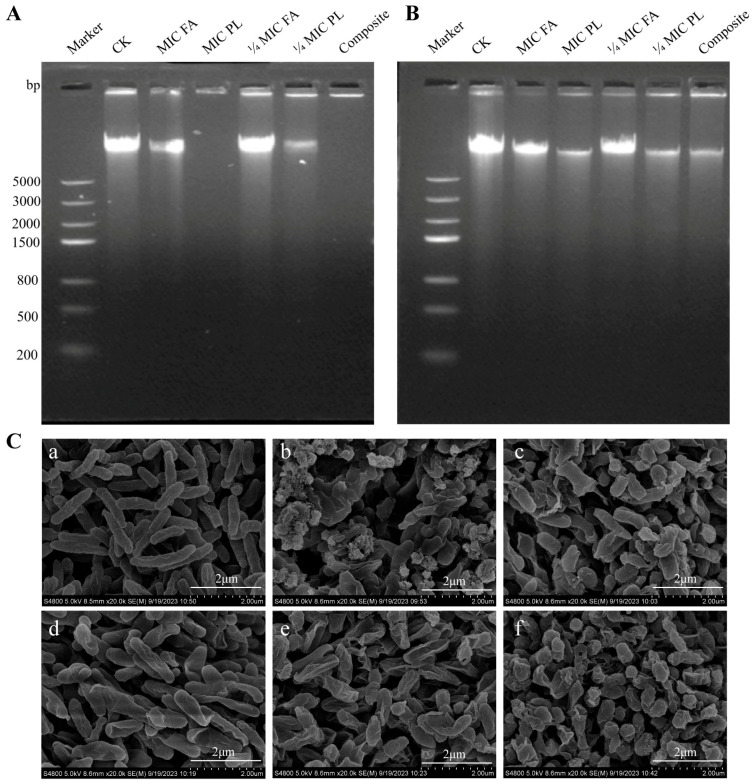
(**A**) Effect of FA and PL on the genomic DNA; (**B**) effect of FA and PL on the genomic DNA in vivo; (**C**) cell morphology of *S. putrefaciens*. (**a**): Control group; (**b**): MIC FA; (**c**): MIC PL; (**d**): 1/4 MIC FA; (**e**): 1/4 MIC PL; (**f**): 1/4MIC FA + 1/4 MIC PL.

The morphological changes of *S. putrefaciens* treated with FA and PL were observed by SEM (Figure 5C) on the smooth cell surface. Changes in cell morphology suggest harm to the cell wall and membrane, which could lead to cell inactivation or death [16]. The cell wall and membrane are essential for maintaining cell shape and providing a stable internal environment for cell development and chemical processes [16]. Cells treated with MIC FA and MIC PL exhibited severe rupture, and cells stuck together, suggesting that cytoplasmic material might be released. Cells treated with 1/4 MIC FA and 1/4 MIC PL also exhibited sunken cell surfaces and shrinking cells with irregular edges, although some cells still maintained an intrinsic rod-like morphology. Similar bacterial cell damage in *S. putrefaciens* has been observed after treatment with different concentrations of PL [11]. In addition, the 1/4 MIC FA and 1/4 MIC PL treatments induced dramatic deformation and cracking in *S. putrefaciens* compared with individual utilization, which resulted in the significant release of more cytoplasmic materials.

### 3.5. Effect of FA and PL on the Genomic DNA of S. putrefaciens

DNA is one of the essential genetic materials in living organisms, serving as a macromolecule indispensable for the normal growth, development, and functioning of these organisms [32]. Any reduction in DNA content or damage to DNA can affect the normal expression of genes, leading to the blockage of intracellular enzyme and receptor protein synthesis, ultimately resulting in the death of the organism [32]. PL can penetrate cells and interact with DNA, thereby influencing the synthesis of biomolecules [33,34]. As shown in Figure 5A, the DNA bands in the control group were the brightest and most abundant. The bands in the 1/4 MIC FA were slightly weakened compared to those in the control group, while the DNA bands in the 1/4 MIC PL group were significantly reduced in brightness and intensity compared to the MIC FA group. The bands in the other treatment groups were dispersed and disappeared, indicating that PL has a strong degradation effect on the DNA of *S. putrefaciens*, with the intensity of degradation being significantly higher than that of FA. In addition, the DNA bands in the combination of 1/4 MIC FA and 1/4 MIC PL were also dispersed, suggesting an enhanced DNA degradation effect from the combination.

In vitro experiments can demonstrate whether a preservative adversely affects DNA, but its impact in vivo is often modulated by the body’s inherent defense and protective mechanisms [33]. Therefore, the effect of different concentrations and types of preservatives on the DNA of *S. putrefaciens* in vivo was examined. As shown in Figure 5B, the DNA bands of the control group were bright. Following treatment with FA and PL, the brightness of the DNA bands of *S. putrefaciens* decreased across all groups, with the most significant reductions observed in the composite group, MIC PL, and 1/4 MIC PL.

Combined experimental analyses of FA and PL on the in vitro DNA of *S. putrefaciens* pombe revealed that DNA degradation in vivo was significantly lower compared to in vitro conditions. This difference may be attributed to the fact that the preservative must penetrate the bacterial cell wall and membrane, which serve as the first line of defense in vivo, resulting in an intracellular concentration of the preservative insufficient to induce complete DNA degradation [32]. In conclusion, findings from both in vitro and in vivo experiments suggest that FA and PL can disrupt the normal DNA anabolism process, demonstrating a synergistic effect in inducing DNA damage.

### 3.6. Effects of PAW, PL, and FA on Quality of Crayfish

#### 3.6.1. Color Difference Analysis

Color represents a primary and direct parameter for evaluating visual quality, playing a pivotal role in shaping consumer perceptions of the overall appearance and acceptability of seafood during purchasing decisions [35]. As shown in Table 1, the L* values of crayfish in all treatment groups exhibited a decreasing trend during storage, which may be attributed to the oxidation of myoglobin to metmyoglobin, leading to the darkening of the crayfish meat over time. The a* values of crayfish meat exhibited an overall increasing trend, with the blank control group showing the most rapid growth in a* values. Notably, the b* values of the control group (CK) showed the most significant increase compared to other treatment groups.

#### 3.6.2. Bacteriostatic Analysis

**Figure 6:**
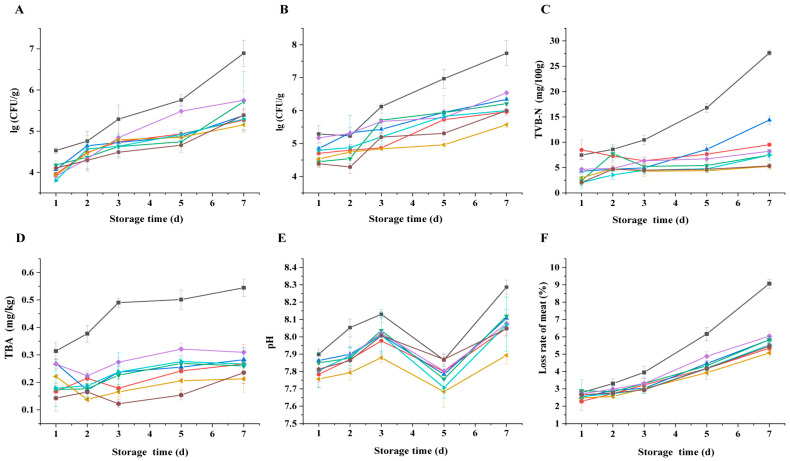
(**A**) Total viable counts (TVCs) with FA, PL, and PAW treatments of the crayfish. (**B**) *S. putrefaciens* bacteriostatic analysis with FA, PL, and PAW treatments of the crayfish. Effect of FA, PL, and PAW on the preservation of crayfish. (**C**) Effect of FA and PL on the preservation of crayfish. Total volatile base nitrogen (TVB-N); (**D**) thiobarbituric acid reactive substance (TBA); (**E**) pH analysis; (**F**) juice loss rate of meat. The data are expressed as mean values ± standard deviation (SD).

TVC is an important index of crayfish freshness, reflecting the antibacterial effects of FA and PL in crayfish. Consequently, the TVC of crayfish contaminated with *S. putrefaciens* was evaluated at 4 °C. As shown in Figure 6A, the TVC in all groups increased in a time-dependent manner. The CK exhibited faster growth, with TVC reaching 6.892 log_10_ CFU/g on d 7. At this point, the TVC of crayfish exceeded 6.0 log_10_ CFU/g, resulting in decayed and inedible crayfish meat. The growth rate of TVC in the PAW + PL-FA group was substantially slower than other groups on d 7. Li et al. [11] also reported that the combination of PL and gallic acid reduced total viable counts compared to the control and extended the shelf life of raw sea bass. The results indicate that the combination of preservatives and PAW treatment can significantly inhibit the growth of microbial colonies in crayfish, with its inhibitory effect being higher than that in the untreated group.

#### 3.6.3. *S. putrefaciens* Bacteriostatic Analysis

The deterioration of frozen foods typically correlates with the proliferation and metabolic activities of psychrophilic and specific spoilage bacteria [36]. *S. putrefaciens* is a predominant spoilage organism commonly found in aquatic products, possessing a potent ability to induce spoilage, thereby influencing the organoleptic quality of crayfish. As shown in Figure 6B, crayfish in all treatment groups showed the growth of *S. putrefaciens* during 7 d, and the CK, without any preservation treatment, showed the most rapid growth, reaching 7.738 log_10_ CFU/g on d 7. The growth rate of *S. putrefaciens* in the PL and FA treatment groups was significantly slower than that in the CK. On this basis, the amount of *S. putrefaciens* in the three groups with PAW was reduced to 5.567, 5.999, and 5.999 log_10_ CFU/g at the end of cold storage, and the number of bacterial colonies was less than that of PAW and the three groups of preservative treatments. It was seen that double immersion treatment could better inhibit the growth and reproduction of *S. putrefaciens*. The combination may enhance the synergistic bacteriostatic effect [11].

#### 3.6.4. TVB-N Analysis

The changes in TVB-N, an essential indicator for assessing the freshness of aquatic products, reflect the level of protein and amine degradation [5]. Research has demonstrated that TVB-N, originating mostly from microbial-driven degradation of aquatic proteins, causes the production of alkaline volatile chemicals such as ammonia and amines. Lower TVB-N levels indicate less degradation of nutrients like tyrosine and methionine, which indicates better nutrient preservation [37,38]. The changes in TVB-N values of crayfish treated with FA and PL are shown in Figure 6C. The rate of increase of the TVB-N value of the CK was significantly higher than that of the other treatment groups. The initial TVB-N value of crayfish was 7.467 mg/100 g, reaching the highest value of 27.627 mg/100 g after 7 d. Tang et al. also reported that the TVB-N value of the crayfish increased with time, and the initial TVB-N value was 8.00 mg/100 g [5]. After seven days of storage, the TVB-N value of the CK crayfish exceeded 20 mg/100 g, the national requirement of Chinese hygienic standards [21]. During the entire storage period, the TVB-N values in the FA, PL, and PL-FA groups were significantly lower than those in the CK, with the PL-FA group exhibiting the lowest TVB-N value. Considering the dosage and cost of preservatives, the combined PAW+ treatment demonstrates superior effectiveness. The dual action of the preservative and low-temperature plasma-activated water effectively inhibits bacterial propagation and reduces the release of amino groups from amino acids [38].

#### 3.6.5. TBA Analysis

TBA values typically indicate the level of lipid oxidation in aquatic products during storage, which is primarily caused by the auto-oxidation of polyunsaturated fatty acids [5]. Lipid oxidation can produce unpleasant odors and induce rancidity and generate potential precursors or catalysts for reactive oxygen species, leading to further food deterioration [15]. Throughout the storage period, the TBA value in the CK consistently increased and remained considerably higher than in the FA, PL, and PL-FA groups (Figure 6D). Individually, both PL and FA effectively controlled TBA levels. It may be because the PL or FA can inhibit lipase activity, reduce the production of free fatty acids, and reduce the rate of fat oxidation. Furthermore, when PAW is employed in conjunction with preservatives, the TBA values exhibited a reduction compared to both the PAW and preservative groups individually. PAW contains oxidative active particles [19], which can oxidize and decompose unsaturated fatty acids in crayfish meat, leading to an increase in TBA. The preservative combined with PAW immersion treatment significantly inhibited the increase in TBA levels in crayfish, suggesting synergistic effects of PL and FA on preservation in crayfish. And the TBA value during refrigeration was much less than the maximum limit of 1.0 mg/kg.

#### 3.6.6. pH Analysis

pH serves as a comprehensive indicator reflecting the freshness of aquatic products during the early stages of deterioration [39,40]. As shown in Figure 6E, the pH of crayfish in all treatment groups exhibited a pattern of increasing, decreasing, and finally increasing with the extension of storage. The initial decline in freshness could be attributed to glycolysis, which results in glycogen degradation in the body to produce lactic acid while consuming ATP to generate inorganic phosphate [41]. The increase in pH can be attributed to the action of microorganisms and endogenous enzymes, resulting in the breakdown of proteins and nitrogenous substances into alkaline substances such as ammonia and trimethylamine. The pH value is influenced by multiple factors and cannot serve as a definitive indicator of crayfish quality changes. Therefore, it is essential to integrate additional parameters to comprehensively evaluate the preservation efficacy of aquatic products [5,13].

#### 3.6.7. Juice Loss Rate of Meat Analysis

The juice loss rate of meat serves as an indicator of the water-holding capacity of meat products. An increase in the juice loss rate reduces the quality of aquatic products and diminishes their weight, leading to economic losses [42]. As shown in Figure 6F, with prolonged cold storage time, the water fluidity within the tissue structure of crayfish increases, weakening the water-holding capacity and elevating the juice loss rate across all treatment groups. The rate of crayfish juice loss in the CK increased most rapidly, from 2.77% on d 1 of refrigeration to 9.068% on d 7, showing the most significant increase. Among them, the combination of PAW and preservative had the best effect; PAW and preservative have an antibacterial effect and inhibit enzyme activity. The rise in the juice loss rate is attributed to the proliferation of microorganisms and protein oxidation, contributing to the accelerated juice loss.

## 4. Conclusions

The combination of 1/4 MIC FA and 1/4 MIC PL demonstrated a synergistic antibacterial mechanism effect against *S. putrefaciens*. It accelerated cell membrane damage and promoted the leakage of intracellular contents. This combination facilitated the entry of FA and PL into the cells, disrupting DNA expression and ultimately leading to cell death. Furthermore, preservatives combined with PAW effectively suppressed microbial proliferation and delayed protein and fat oxidation. This method helps in maintaining meat elasticity and moisture, thereby augmenting the taste and quality of crayfish. Therefore, the combination of FA and PL, combined with PAW, could represent a novel strategy for controlling crayfish storage. In addition, before their application in food products, the combined effects of FA and PL with PAW require thorough investigation across a broad range of microbes.

## Figures and Tables

**Table 1:** The crayfish color difference experiment results.

Group		1 d	2 d	3 d	5 d	7 d
L*	CK	81.95 ± 1.08 ^d^	80.07 ± 0.57 ^b^	79.63 ± 1.86 ^b^	78.01 ± 2.25 ^c^	72.93 ± 2.22 ^c^
	FA	82.88 ± 0.83 ^cd^	81.85 ± 0.96 ^ab^	81.27 ± 1.34 ^ab^	79.93 ± 0.62 ^b^	78.68 ± 0.38 ^ab^
	PL	85.14 ± 0.46 ^ab^	82.97 ± 0.58 ^a^	81.03 ± 1.07 ^ab^	80.09 ± 1.14 ^ab^	77.72 ± 0.12 ^ab^
	PL-FA	85.12 ± 0.97 ^ab^	82.15 ± 0.31 ^ab^	81.53 ± 0.22 ^ab^	80.06 ± 0.18 ^ab^	77.56 ± 1.67 ^ab^
	PAW	84.02 ± 0.36 ^bc^	81.79 ± 2.20 ^ab^	80.84 ± 1.46 ^ab^	79.11 ± 0.69 ^bc^	76.44 ± 0.13 ^b^
	PAW-FA	84.57 ± 0.49 ^ab^	83.66 ± 1.31 ^a^	82.23 ± 0.25 ^a^	80.74 ± 0.85 ^ab^	78.85 ± 1.19 ^a^
	PAW-PL	85.58 ± 0.68 ^a^	84.12 ± 0.03 ^a^	82.54 ± 1.55 ^a^	81.97 ± 0.22 ^a^	79.36 ± 0.85 ^a^
	PAW + PL-FA	84.10 ± 0.70 ^bc^	83.21 ± 2.10 ^a^	82.01 ± 1.40 ^ab^	80.70 ± 0.67 ^ab^	78.01 ± 0.36 ^ab^
a*	CK	3.03 ± 0.17 ^a^	3.67 ± 0.29 ^a^	5.12 ± 0.13 ^a^	7.05 ± 0.08 ^a^	8.07 ± 0.18 ^a^
	FA	2.20 ± 0.12 ^bcd^	2.38 ± 0.72 ^bc^	3.40 ± 0.26 ^c^	3.44 ± 0.13 ^cd^	3.54 ± 0.15 ^d^
	PL	1.96 ± 0.35 ^cde^	2.08 ± 0.12 ^c^	3.04 ± 0.08 ^d^	3.69 ± 0.15 ^bcd^	4.08 ± 0.22 ^c^
	PL-FA	2.37 ± 0.17 ^bc^	2.83 ± 0.20 ^b^	3.56 ± 0.20 ^c^	3.78 ± 0.20 ^bc^	4.45 ± 0.28 ^b^
	PAW	2.54 ± 0.33 ^b^	2.89 ± 0.37 ^b^	3.92 ± 0.20 ^b^	4.01 ± 0.09 ^b^	4.27 ± 0.12 ^bc^
	PAW-FA	1.91 ± 0.10 ^de^	2.00 ± 0.32 ^c^	2.43 ± 0.09 ^e^	2.88 ± 0.19 ^e^	3.43 ± 0.09 ^d^
	PAW-PL	1.60 ± 0.26 ^e^	1.83 ± 0.32 ^c^	2.98 ± 0.10 ^d^	3.48 ± 0.32 ^cd^	3.96 ± 0.22 ^c^
	PAW + PL-FA	2.29 ± 0.22 ^bcd^	2.44 ± 0.28 ^bc^	3.46 ± 0.22 ^c^	3.39 ± 0.24 ^d^	4.27 ± 0.10 ^bc^
b*	CK	8.45 ± 0.39 ^a^	8.90 ± 0.31 ^a^	9.28 ± 0.66 ^a^	10.90 ± 0.16 ^a^	12.22 ± 0.18 ^a^
	FA	7.65 ± 0.24 ^bcd^	8.29 ± 0.46 ^ab^	8.60 ± 0.51 ^ab^	9.43 ± 0.65 ^bc^	10.19 ± 0.04 ^b^
	PL	7.65 ± 0.51 ^bcd^	7.94 ± 1.26 ^ab^	9.07 ± 0.19 ^ab^	9.47 ± 0.22 ^bc^	9.97 ± 0.21 ^bc^
	PL-FA	7.97 ± 0.16 ^abc^	8.41 ± 0.55 ^ab^	8.89 ± 0.69 ^ab^	9.70 ± 0.38 ^b^	10.21 ± 0.71 ^b^
	PAW	8.03 ± 0.23 ^ab^	8.29 ± 0.34 ^ab^	8.93 ± 0.55 ^ab^	9.48 ± 0.23 ^bc^	10.65 ± 0.78 ^b^
	PAW-FA	7.44 ± 0.32 ^cd^	8.12 ± 0.48 ^ab^	8.54 ± 0.23 ^ab^	9.22 ± 0.17 ^bcd^	9.90 ± 0.11 ^bc^
	PAW-PL	7.18 ± 0.11 ^d^	7.59 ± 0.53 ^b^	8.41 ± 0.55 ^ab^	8.97 ± 0.31 ^cd^	8.95 ± 0.72 ^c^
	PAW + PL-FA	7.85 ± 0.17 ^bc^	8.16 ± 0.38 ^ab^	8.27 ± 0.39 ^b^	8.61 ± 0.49 ^d^	9.77 ± 0.47 ^bc^

The results are expressed as the means ± standard deviations. Different lowercase letters (a, b, c and d) indicate significant differences among the different groups within the same color parameter (*p* < 0.05).

## Data Availability

Data are contained within the article.

## Abbreviations

The following abbreviations are used in this manuscript:

FA	ferulic acid
PL	ε-polylysine hydrochloride
*S. putrefaciens*	*Shewanella putrefaciens*
FICI	fractional inhibitory concentration index
MIC	Minimum Inhibitory Concentration
PAW	plasma-activated water
TVC	total viable count
TVB-N	total volatile base nitrogen
TBA	thiobarbituric acid value
NPN	N-phenyl-1-naphthylamine
PI	propidium iodide
PBS	phosphate-buffered saline
TSB	Tryptic Soy Broth
PCA	plate count agar
AKP	alkaline phosphatase
SEM	scanning electron microscopy
